# Dietary polyamines promote intestinal adaptation in an experimental model of short bowel syndrome

**DOI:** 10.1038/s41598-024-55258-4

**Published:** 2024-02-26

**Authors:** Naoya Kasahara, Takumi Teratani, Shinichiro Yokota, Yasunaru Sakuma, Hideki Sasanuma, Yasuhiro Fujimoto, Tetsuo Ijichi, Taizen Urahashi, Hideyuki Yoshitomi, Joji Kitayama, Naohiro Sata

**Affiliations:** 1 Department of Surgery, Jichi Medical University, Shimotsuke, Japan; 2 Division of Translational Research, Jichi Medical University, Shimotsuke, Japan; 3 Department of Transplant Surgery, Nagoya University Hospital, Nagoya, Japan; 4 Department of Surgery, Dokkyo Medical University Saitama Medical Center, Koshigaya, Japan

**Keywords:** Diseases, Gastroenterology

## Abstract

Intestinal adaptation does not necessarily recover absorptive capacity in short bowel syndrome (SBS), sometimes resulting in intestinal failure-associated liver disease (IFALD). Additionally, its therapeutic options remain limited. Polyamines (spermidine and spermine) are known as one of the autophagy inducers and play important roles in promoting the weaning process; however, their impact on intestinal adaptation is unknown. The aim of this study was to investigate the impact of polyamines ingestion on adaptation and hepatic lipid metabolism in SBS. We performed resection of two-thirds of the small intestine in male Lewis rats as an SBS model. They were allocated into three groups and fed different polyamine content diets (0%, 0.01%, 0.1%) for 30 days. Polyamines were confirmed to distribute to remnant intestine, whole blood, and liver. Villous height and number of Ki-67-positive cells in the crypt area increased with the high polyamine diet. Polyamines increased secretory IgA and mucin content in feces, and enhanced tissue Claudin-3 expression. In contrast, polyamines augmented albumin synthesis, mitochondrial DNA copy number, and ATP storage in the liver. Moreover, polyamines promoted autophagy flux and activated AMP-activated protein kinase with suppression of lipogenic gene expression. Polyamines ingestion may provide a new therapeutic option for SBS with IFALD.

## Introduction

Short bowel syndrome (SBS) is defined as a state of the loss of intestinal absorptive capacity due to surgical resection, congenital defect, or disease. Its symptoms include diarrhea, malnutrition, and dehydration. Survival and reasonable quality of life for SBS patients depend on adaptation of the remnant bowel, as well as medical, nutritional, and pharmaceutical therapies provided by an intestinal rehabilitation program (IRP).

Patients who are unable to increase their oral intake or absorb sufficient nutrients require long-term parenteral nutrition (PN)^1^. Although PN is life-saving for patients with SBS, attention needs to be paid to potentially fatal complications such as intestinal failure-associated liver disease (IFALD), catheter-related bloodstream infection, and thrombosis^2^. IFALD caused by long-term administration of PN may result in cholestasis, steatohepatitis, fibrogenesis, and progression to end-stage liver disease^3^. PN-related biliary cirrhosis in patients with severe intestine failure (IF) is a major indication for small bowel or multi-visceral transplantations^4^. Recently, hormonal therapy of glucagon-like peptide-2 (GLP-2) has started to play a role in intestinal adaptation to allow a substantial proportion of patients to be weaned off PN^5^; however, its high cost may limit the clinical availability. New therapeutic modalities that enhance intestinal absorption of the remnant bowel and enable patients to be weaned off PN support are thus needed.

Polyamines consisting of putrescine (Put), spermidine (Spd), and spermine (Spm) are ubiquitous organic cations that are essential for cell growth and viability in mammals. Because they bind to cellular anions such as DNA, RNA, proteins, and phospholipids, they play important roles as modulators of DNA stability, RNA transcription, and protein synthesis. Dietary polyamine supplementation has recently emerged as a promising strategy to increase longevity and delay aging^6,7^, attributed to its induction of autophagy^8^.

Polyamines stimulate gut epithelial renewal by regulating the expression of various genes. An increase in polyamine levels promotes the expression of growth-promoting genes by activating their gene transcription^9^, whereas it inhibits the expression of growth-inhibiting genes post-transcriptionally^10^. Spermidine serves as the unique substrate for the hypusination of a lysine residue in eukaryotic initiation factor 5A (EIF5A). Hypusinated EIF5A modulates protein synthesis through involvement in translation initiation, elongation, and termination^11^. Previous studies demonstrated that spermidine treatment restored hypusinated EIF5A, protein synthesis, and mitochondrial fatty acid oxidation in an animal model of non-alcoholic steatohepatitis (NASH)^12^.

We reported that oral polyamine administration ameliorated hepatic ischemic reperfusion injury and promoted regeneration in a rodent model mimicking living donor partial liver transplantation^13,14^. Moreover, we demonstrated that a high-polyamine diet (PD) increased ATP content throughout the body, suggesting that polyamines enhanced ATP production in mitochondria^15^. However, little is known about oral polyamine intake’s impact on adaptation after massive intestine resection and lipid metabolism in liver. Here, we show that dietary polyamines promote intestinal adaptation in a rat SBS model via a GLP-2-independent mechanism and enhance autophagy as well as suppress lipogenic gene expression in liver.

## Results

### Polyamines distribution to tissues and enhanced adaptation in SBS rat model

**Figure 1:**
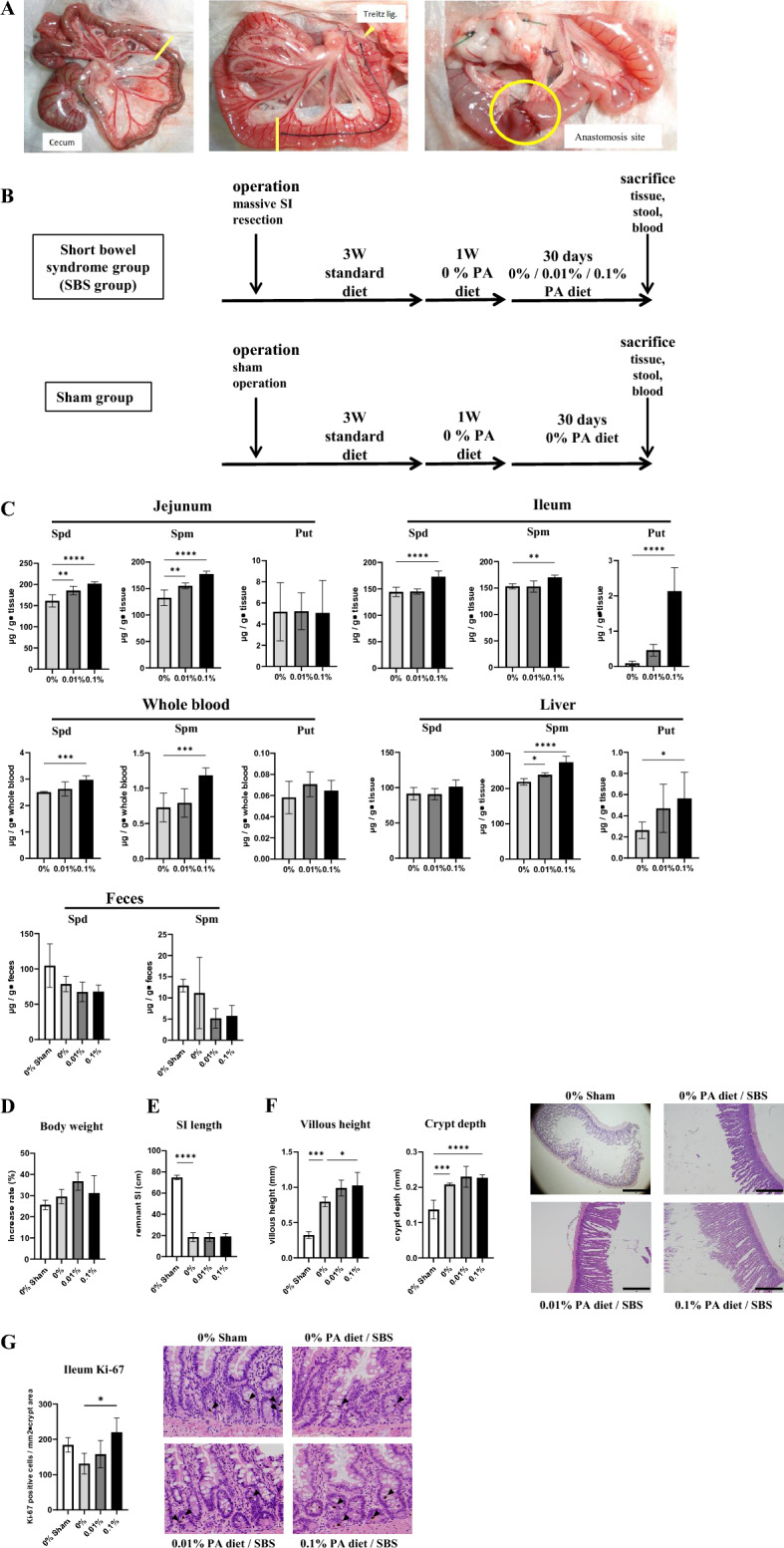
Distribution of orally administered polyamines to the target organs and effect of polyamines on intestinal adaptation of remnant ileum. **(A)** Experimental protocol. **(B)** Our model of massive intestinal resection. **(C)** The contents of spermidine (Spd), spermine (Spm), and putrescine (Put) in remnant jejunum, remnant ileum, whole blood, liver, and feces were measured in rats with massive intestinal resection fed respective polyamine concentration diets. **(D)** Rate of body weight gain from the beginning of feeding on respective polyamine concentration diets. **(E)** The length of small intestine. **(F)** From H&E-stained slides, villous height and crypt depth of ileum were determined using NIH ImageJ software. Representative images are shown. Scale bar, 1 mm. **(G)** Quantification of Ki-67-positive cells in crypt area of ileum. Representative ileum sections stained by Ki-67. Arrowheads indicate Ki-67-positive nuclei of crypt cells (original magnification, ×200; scale bars = 100 μm). n = 6–7 rats (n = 3 fecal samples of sham group). Data are presented as mean ± SD. Results of one-way ANOVA are represented as follows: *P < 0.05; **P < 0.01; ***P < 0.001; ****P < 0.0001.

Three weeks of feeding on a standard diet after surgery was intended to avoid the negative impact of a polyamine-deficient diet on the recovery period. The subsequent week of 0% PD was designed to deplete polyamine storage at the chronic stage, to illuminate the effect of feeding on the respective PD (Fig. 1A,B). To confirm the polyamines distribution upon ingestion, polyamines content was measured by HPLC. Spd and Spm were distributed to remnant jejunum, remnant ileum, whole blood, and liver in a dietary polyamine concentration-dependent manner, except for Spd in liver (Fig. 1C). In contrast, tissue Put contents were increased in ileum and liver in a similar fashion. Polyamine catabolism is known to involve the back conversion pathway, in which spermine or spermidine is acetylated and subsequently oxidized to produce spermidine or putrescine, respectively. Because the diet used here does not contain Put, the above-mentioned increases may have been attributable to the back conversion pathway. The sham group had an average preoperative weight of 222 g, and their weight increased by an average of 22 g, representing a 9.7% increase at 1 week after surgery. While the SBS group had an average preoperative weight of 217 g, and their weight decreased by an average of 6 g, representing a 2.4% decrease. No significant difference in the rate of body weight increase was found among the groups (Fig. 1D). The remnant intestine in the SBS groups did not increase in length regardless of the growth in body size, while it did increase in the sham group (Fig. 1E). The villous height and crypt depth of remnant ileum were increased in the SBS groups compared with those in the sham group (Fig. 1F). The villous height was increased in a dietary polyamine concentration-dependent manner in the SBS groups. The number of Ki-67-positive cells in the ileum crypt area decreased with intestine resection, but significantly increased in the SBS group fed a high PD (Fig. 1G), implying that polyamines promote adaptation to achieve gut epithelial renewal.

**Figure 2:**
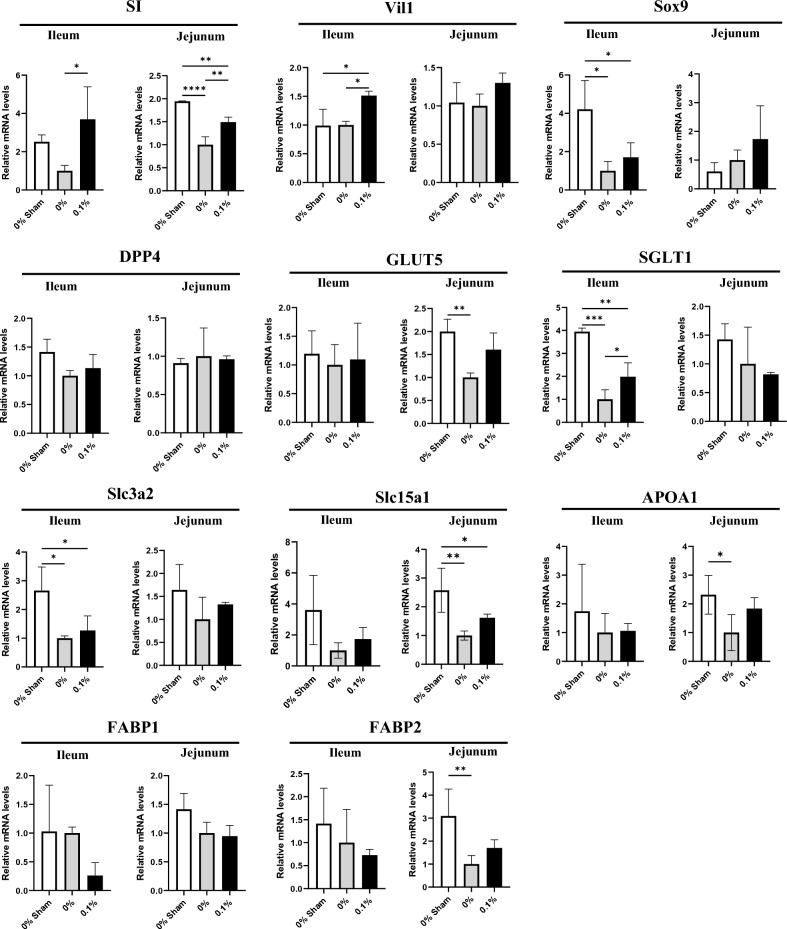
Effects of polyamines on marker gene mRNA expression in ileum. Relative mRNA expression of differentiation markers, sugar transporters, amino acid transporters, and fatty acid transporters in intestine. GAPDH was used as a loading control. The mean expression level of 0% PD-fed rats was defined as 1.0. Data are presented as mean ± SD. Results of one-way ANOVA are represented as follows: *P < 0.05; **P < 0.01; ***P < 0.001; ****P < 0.0001.

To assess polyamine’s impact on mRNA expression in remnant ileum, RT-qPCR was performed (Fig. 2) on the following mRNA: SI and Vil1 as marker genes associated with absorptive epithelium; Sox9 and DPP4 for differentiation; GLUT5 and SGLT1 for monosaccharide transporter; Slc3a2 and Slc15a1 for amino acid transporter; and APOA1, FABP1, and FABP2 for fatty acid transporter. The mRNA expression of ileal Sox9, SGLT1, and Slc3a2 and jejunal SI, GLUT5, Slc15a1, APOA1, and FABP2 was significantly downregulated in the 0% PD group compared with that in the sham group. The expression levels of SI in both ileum and jejunum, Vil1 in ileum, and SGLT1 in ileum significantly increased in the 0.1% PD-fed group compared with those in the 0% PD-fed group.

### Polyamines supplementation enhances gut barrier function

**Figure 3:**
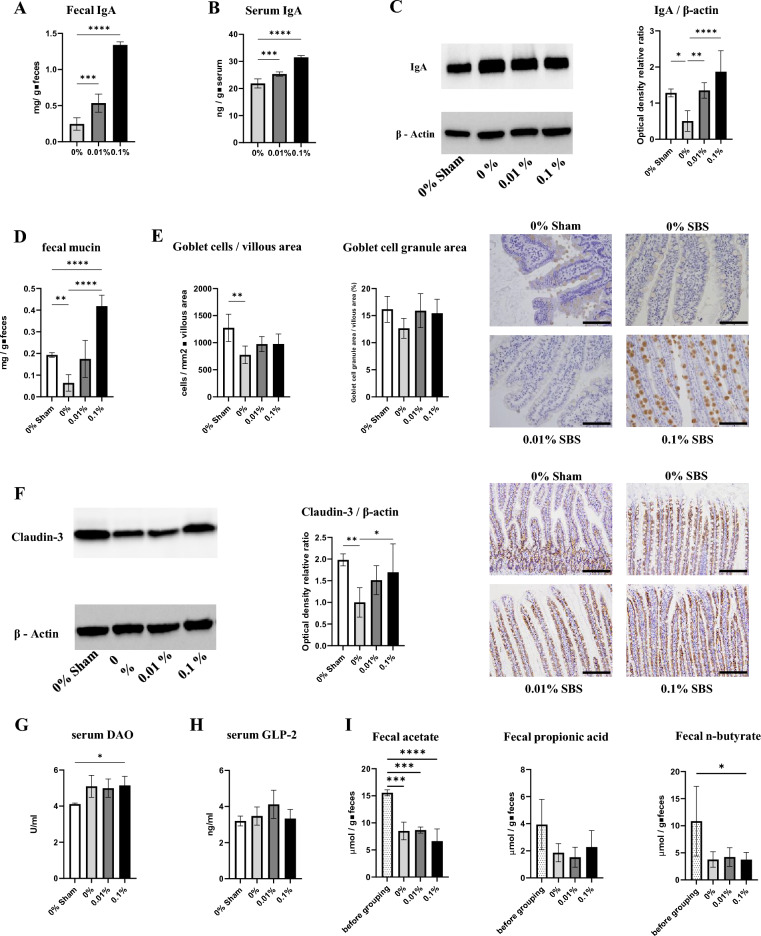
Polyamines enhance mucosal defense factors in rats with massive intestinal resection. **(A-B)** Fecal and serum secretory IgA was measured using ELISA. n = 5–7/group. **(C)** IgA in ileum tissue was assessed using western blotting. n = 4–6/group. **(D)** Fecal mucin was measured using a fluorometric assay. n = 4–6/group. **(E)** Representative images of ileal villus showing immunostaining by Muc2 (original magnification, ×400; scale bars = 200 μm). Left graphs show the number of goblet cells per unit villous area and the size of goblet cell secretion granule. **(F)** The expression of Claudin-3 in the ileum tissue was measured by western blot analysis. Representative images of ileal villus showing immunostaining by Claudin-3 (original magnification, ×200; scale bars = 100 μm). **(G)** Serum DAO was measured by ELISA. n = 5–7/group. **(H)** Serum GLP-2 was measured by ELISA. n = 5–7/group. **(I)** Fecal short-chain fatty acid (SCFA) content was measured using high-performance liquid chromatography. n = 3–6/group. Data are presented as mean ± SD. Results of one-way ANOVA are represented as follows: *P < 0.05; **P < 0.01; ***P < 0.001; ****P < 0.0001.

Fecal and serum IgA increased with dietary polyamines supplementation in the SBS groups (Fig. 3A,B). Interestingly, ileum tissue IgA decreased through massive intestine resection, while it increased to that in the sham group upon feeding on a high PD (Fig. 3C). Fecal mucin content showed a trend similar to that of tissue IgA content (Fig. 3D). Although the number of goblet cells in the villous area decreased with intestine resection, polyamines supplementation did not alter this among the SBS groups (Fig. 3E). The area of secretory granules per goblet cell did not differ significantly among the groups (Fig. 3E), whereas Muc2 staining of secretory granules showed evidently strong staining in the 0.1% PD-fed group compared with the findings in the other groups (Fig. 3E). Expression of the tight junction protein Claudin-3 in ileum decreased in the SBS groups, whereas it increased in a dietary polyamine concentration-dependent manner (Fig. 3F). Intriguingly, Claudin-3 immunostaining in ileum revealed potent expression at the villous root in the sham group and evenly distributed expression on villi in the SBS groups. The serum level of DAO was reported to be associated with the structure and integrity of the small intestinal mucosa^16^. The level of serum DAO increased in the 0.1% PD-fed SBS group compared with that in the sham group (Fig. 3G). No significant difference was observed in serum GLP-2 among the groups after overnight fasting (Fig. 3H). Meanwhile, fecal acetate content was significantly reduced in the SBS groups compared with that at baseline (before grouping) (Fig. 3I). There were similar trends in fecal propionate content, with no significant differences observed. Fecal content of n-butyrate was significantly lower only in the 0.1% PD-fed group compared with that at baseline.

### Polyamines promotes liver autophagy to improve energy status and alter lipid metabolism

**Figure 4:**
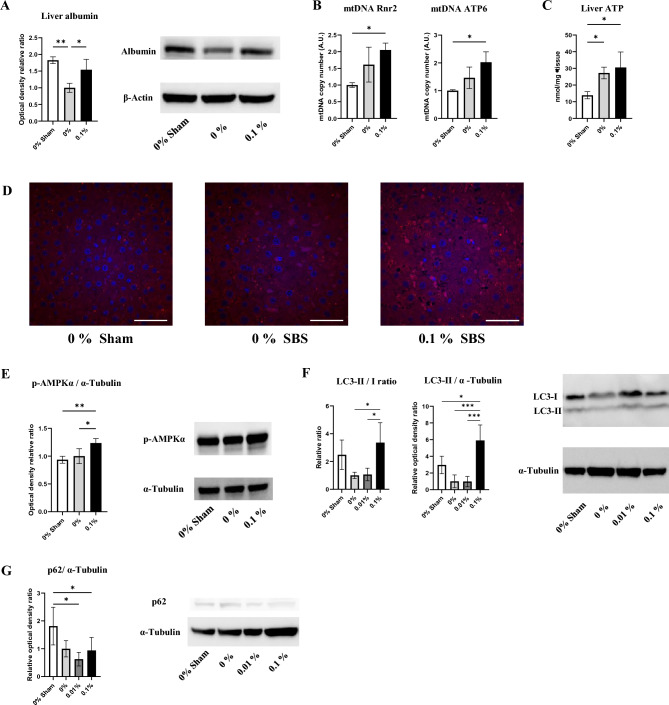
Polyamines improve hepatic nutritional status and enhance autophagy flux. **(A)** Albumin in liver tissue was assessed using western blot. n = 4–6/group. **(B)** Relative hepatic mtDNA copy number. n = 4–7/group. **(C)** Liver tissue ATP content. **(D)** Representative images of liver fluorescent immunohistochemistry for mitochondrial Hsp60 (red) and DAPI (blue) in liver sections (original magnification, ×600; scale bars = 50 μm). **(E)** Phosphorylation of AMPKα at Thr172 in liver. n = 4–6/group. Representative western blots are shown (right). **(F)** Representative western blot for LC3 in liver (right). Upper band corresponds to Lc3-I and lower band to LC3-II, with α-Tubulin as the loading control. Quantification of LC3-II and LC3-I protein abundance relative to α-Tubulin, n = 4–6/group. **(G)** The expression of p62 in liver tissue was measured by western blot analysis. Representative western blot for p62 in liver (right). n = 4–6/group. Data are presented as mean ± SD. Results of one-way ANOVA are represented as follows: *P < 0.05; **P < 0.01; ***P < 0.001.

**Figure 5:**
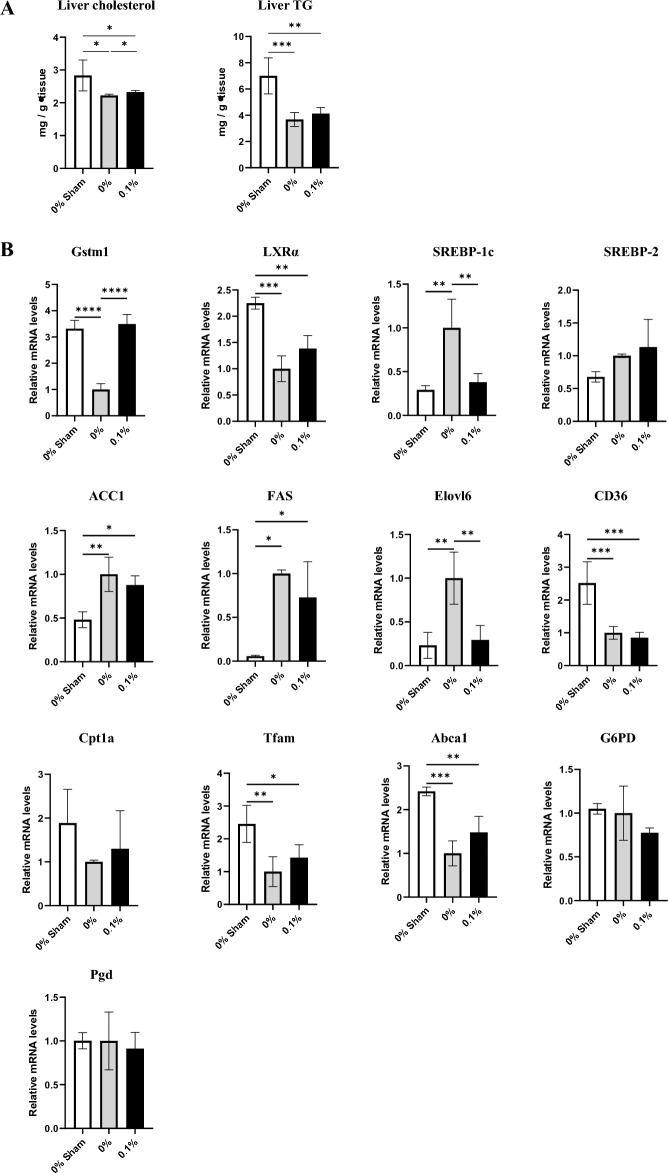
Polyamines suppress the expression of lipogenic genes. **(A)** Liver tissue cholesterol content. n = 3–5/group. **(B)** Liver tissue triglyceride content. n = 3–5/group. **(C)** Relative mRNA expression of oxidative stress-related genes, and lipid and glucose metabolism-related genes in liver. n = 4–6/group. β-Actin was used as a loading control. The mean expression level of 0% PD-fed rats was defined as 1.0. Data are presented as mean ± SD. Results of one-way ANOVA are represented as follows: *P < 0.05; **P < 0.01; ***P < 0.001; ****P < 0.0001.

Western blot analyses revealed that liver albumin content was reduced by massive intestine resection and recovered in the 0.1% PD-fed group compared with that in the sham group (Fig. 4A). mtDNA copy number and ATP content in liver tissue were significantly increased in the 0.1% PD-fed group compared with those in the sham group (Fig. 4B,C). Staining for the mitochondrial protein Hsp60 revealed greater intensity in the 0.1% PD-fed group, suggesting increased mitochondrial volume and density (Fig. 4D). This was consistent with the above-mentioned increase in relative mitochondrial (mt)DNA copy number (Fig. 4B). Phosphorylated AMPKα significantly increased in the 0.1% PD-fed group compared with those in the sham group and 0% PD-fed group (Fig. 4E). Meanwhile, the LC3-II/I ratio in liver was significantly increased in the 0.1% PD-fed group compared with that in the 0% PD-fed group, implying enhanced autophagy flux by polyamines supplementation (Fig. 4F). LC3-II expression was significantly increased in the 0.1% PD-fed group compared with that in the other groups, implying an increased number of autophagosomes (Fig. 4F). Finally, p62 expression exhibited a significant decrease in the 0% and 0.1% PD-fed groups compared with that in the sham group (Fig. 4G). Liver cholesterol and triglyceride contents were decreased in the SBS groups compared with those in the sham group (Fig. 5A). Liver cholesterol content was increased in the 0.1% PD-fed group compared with that in the 0% PD-fed group. To assess polyamine’s effect on mRNA expression of lipid- and glucose metabolism-related genes in liver, RT-qPCR was performed (Fig. 5B). Glutathione S-transferase (Gst) is a pivotal intracellular antioxidant and antidote for carcinogens, drugs, and environmental toxins. mRNA expression of Gstm1 in liver was decreased in the 0% PD-fed SBS group and recovered in the 0.1% PD-fed group. The expression levels of SREBP-1c, which is a master regulator of the lipogenic pathway, and Elovl6 were increased in the 0% PD-fed SBS group and decreased in the 0.1% PD-fed group. Significantly decreased expression of the mRNA levels of LXRα, CD36, Tfam, and Abca1 was identified in the SBS groups compared with that in the sham group. Meanwhile, there was significantly increased mRNA expression of ACC1 and FAS in the SBS groups compared with that in the sham group. The expression levels of G6PD and Pgd, which are key rate-limiting enzymes of the pentose phosphate pathway, did not show any significant changes among the groups.

## Discussion

Polyamine is known to have beneficial effects on intestinal maturation in weaning animals^17^ and prevent non-alcoholic steatohepatitis (NASH) progression by restoring mitochondrial function^12^. Here, we extend these benefits to an SBS rodent model, resulting in enhanced adaptation and gut barrier function as well as liver autophagy promotion. Furthermore, polyamines supplementation increased hepatic albumin synthesis and ATP storage along with suppressing lipid synthesis-related genes and AMPK activation. Therefore, polyamine ingestion is potentially therapeutic for SBS patients with IFALD.

Breast milk and infant formula serves as the initial dietary sources of polyamine exposure. We measured the polyamine content of enteral nutrition products that have been domestically approved by supervisory authority and clinically used as infant formulas in Japan, but none of them proved to contain polyamines (data not shown). Although there is variability in reports, it is well-established that breast milk contains a substantial amount of polyamines. According to the previous report^18^, full-term breast milk contained 0.824 nmol/ml of putrescine, 4.578 nmol/ml of spermidine, 1.735 nmol/ml of spermine, respectively. In the same report, one of the products commonly used as a first infant formulas available in Sweden proved to contain 3.880 nmol/ml of putrescine, 2.265 nmol/ml of spermidine, 0.363 nmol/ml of spermine, respectively.

The present study showed that supplemented Spd and Spm are absorbed from the intestinal lumen into systemic circulation, enabling them to reach their target tissue, such as remnant intestine, liver, and whole blood. Although polyamine’s half-life in blood after oral administration is relatively short^13^, it was distributed mostly in a concentration-dependent manner despite overnight fasting. Notably, Put content in remnant ileum and liver tissue was increased in food containing Spd and Spm in a concentration-dependent manner. This can be potentially explained by the enzymatic reverse conversion of polyamine oxidase (PAO)^19^, although the precise mechanism remains unclear. Meanwhile, fecal polyamine content showed no difference regardless of small intestine resection and food polyamine content. This is consistent with the fact that most polyamines taken orally are absorbed from the small intestine.

Adaptation after massive intestine resection is defined as the recovery of absorption capacity by compensatory mucosal proliferation^20^. Analogously to the maturation-promoting effect at weaning, this study showed that villous height and crypt depth were increased in the SBS groups compared with those in the sham group, which can be explained as adaptation, and they showed significant increases in a dietary polyamine concentration-dependent manner in the SBS groups. Similar findings were made for the number of cells positive for Ki-67, a marker of proliferation, in the crypt area in the SBS group. Unexpectedly, short bowel models had a lower Ki-67 positivity rate than that of the sham group. In terms of adaptation, this is contrary to the literature for short bowel models. However, the timing of the measurements was about two months post-surgery, which correspond to the chronic phase as short bowel syndrome. We speculate that the decrease in the number of Ki-67 positive cells in the short bowel group reflects the decrease in regeneration capacity of the intestinal epithelium affected by host nutritional status. If the experiment had ended in the early postoperative period, the results might have been as described in the literature. These results suggest that oral polyamine intake stimulates gut epithelial renewal to promote adaptation and increase remnant small bowel surface area to enhance digestive absorption capacity. Interestingly, while intestine length markedly increased with increasing body length in the sham group, there was little increase in the SBS groups regardless of dietary polyamine concentration, implying that polyamines did not affect intestinal lengthening in SBS.

Secretory IgA (sIgA), mucin, and Claudin-3 play critical roles in mucosal barrier function to prevent bacterial translocation^21–23^. Importantly, the present study showed that tissue sIgA, Claudin-3, and fecal mucin were decreased with SBS and restored by 0.1% PD supplementation. sIgA regulates the exclusion of environmental antigens such as bacteria, viruses, and toxins from intestinal mucosa^24^. It also presents antigens to induce Th2- or regulatory T-cell-mediated mucosal immune responses and maintains commensal homeostasis by enhancing the crosstalk between bacteria and intestinal mucosa^25^. Mucin is secreted by intestinal goblet cells. It is densely glycosylated and thereby resistant to various degradative enzymes of bacteria or host. It also serves as a storage layer of IgA and bactericidal peptide as well as a fermentation substrate for bacteria^26^. However, the effect of polyamines on sIgA and mucin production is not well documented. The claudin family proteins, which act as a sealing component of tight junction (TJ), form pores between epithelial cells to regulate paracellular fluid and ion movement^27^. A previous report revealed that polyamines affect HuR, a kind of RNA-binding protein, its phosphorylation, and its binding affinity^28^. According to the report, polyamines regulate TJ barrier function by altering a specific kind of noncoding RNA association with TJ mRNAs, including Claudin-3, via the control of HuR phosphorylation, resulting in enhanced Claudin-3 expression. Taken together, our results demonstrate that polyamines multi-directionally enhance barrier function in remnant intestine.

Short-chain fatty acids (SCFAs), consisting of acetate, propionate, and butyrate, are metabolites produced by the bacterial fermentation of intestinal dietary fiber^29^. In our study, polyamines supplementation did not alter fecal SCFA content among the SBS groups, whereas fecal acetate content decreased over time after feeding on a diet containing each polyamine concentration. This suggests that polyamines supplementation does not promote bacterial production of SCFAs and that the IgA increase in the SBS group fed 0.1% PD is not attributable to SCFAs.

In 1996, Drucker et al. identified GLP-2 as the multiple proglucagon-derived peptide with the most potent intestinotrophic activity^30^. GLP-2 is secreted from intestinal L cells distributed mainly in the ileum to the colon. It acts through the GLP-2 receptor expressed on cells of the gastrointestinal tract, enteric nervous plexus, and central nervous system^31^. A degradation-resistant GLP-2 analog, teduglutide, was subsequently designed and proven to be effective at increasing fluid and energy absorption, while reducing the need for PN in PN-dependent SBS in the clinical trial^5,32^. Recently, teduglutide was approved for chronic therapy for PN-dependent SBS patients in several countries. In a fashion similar to GLP-2’s intestinal function, our results revealed that polyamines supplementation enhanced gut barrier function and energy absorption, thereby promoting albumin synthesis in liver. Meanwhile clinical study in pediatric intestinal failure has shown that serum GLP-2 levels proportionally increased depending on the length of resected small intestine and increase in serum GLP-2 levels reduced requirement for parenteral support in parallel^33^. Importantly, polyamines supplementation did not increase serum GLP-2 in the SBS groups, implying that polyamines facilitate adaptation via a GLP-2-independent pathway.

IFALD in children presents progressive cholestasis and biliary fibrosis, which are characteristic symptoms in biliary atresia. It occurs in 60% of infants with PN-dependent SBS, 25%–40% of whom progress to liver failure^34^. Meanwhile, in adults, it is characterized by steatohepatitis, and some cases progress to NASH, especially in individuals dependent on prolonged PN administration. Abnormal lipid metabolism is supposed to be involved in IFALD development. In patients with IF, over- or undernutrition as well as essential fatty acid deficiency alters lipid metabolism in liver. In addition, sepsis caused by dysbiosis of intestinal commensal microbiota and hepatocellular disorder caused by decreased portal blood flow potentially contribute to disease development. With the progression of IFALD to irreversible liver failure, combined liver and small intestine transplantation is needed. Recently, fish oil intravenous lipid emulsion (FO ILE)^35^ and mixed oil ILE (MO ILE)^36^ were approved for clinical use in soybean oil-based ILE (SO ILE)-intolerant patients with IFALD; however, only a few case reports have shown the effectiveness of FO ILE and MO ILE for improving IFALD. Thus, another therapeutic approach is required.

Autophagy is a lysosome-dependent catabolic process activated by starvation^37^. It is essential for starvation-induced lipid droplet formation in liver and subsequent ketogenesis for maintaining systemic energy homeostasis^38^. A previous report revealed that Spd post-translationally modifies eIF5A, which is essential for synthesis of the autophagy transcription factor EB (TFEB)^39^. TFEB is required to induce starvation response genes in mammals, and thus its absence results in impaired lipid catabolism. Additionally, a recent study showed that the AMPK–TFEB axis ameliorates fat accumulation upon high-fat diet feeding or in an animal model with nutrient-deprivation-induced steatohepatitis^40^. Our results indicated that polyamines enhanced autophagy flux and promoted albumin synthesis in liver. It also boosted AMPK phosphorylation and inhibited its downstream lipogenic genes, such as SREBP-1c and Elovl6, but not FAS and ACC. These actions could eventually lead to decreased liver triglycerides, which could be revealed by extending the surveillance period after surgery. A low-protein diet has been shown to induce mitochondrial dysfunction, characterized by impairments of oxidative phosphorylation, TCA cycle, and β-oxidation in liver^41^. While liver mtDNA was decreased in a study using liver specimens from NAFLD patients^42^, it increased significantly in the SBS group fed a high-polyamine diet, compared with that in the sham group in the present study. In addition, liver ATP content increased in a similar fashion to that of mtDNA. Taken together, these results suggest that polyamines could enhance mitochondrial respiration through AMPK phosphorylation.

In the present study, resection of approximately two-thirds of the intestine was performed. At the end of the experiment, the length of remnant intestine in the SBS groups corresponded to one-fifth of that in the sham group. Upon converting the length of remnant intestine in the SBS groups to its equivalent in human, it corresponds to approximately 1 m. The severity of SBS in the present animal model is mild, and thus no PN is required for survival, provided that human clinical data also apply here. Therefore, our animal model did not completely simulate human SBS patients with IFALD. Recent studies have revealed that ghrelin, FO ILE, GLP-2, and recombinant human hepatocyte growth factor demonstrated therapeutic effects in a rodent model of IFALD with 80%–90% massive intestine resection receiving catheter replacement in the jugular vein^43–46^. To confirm the beneficial effect of polyamines in IFALD, further investigation using an animal model of more massive intestine resection with PN will be needed.

In conclusion, the present study highlights the novel benefits of polyamines in intestinal adaptation for SBS and associated impaired liver disorder. The finding that polyamines enhanced gut barrier function suggests its possible preventive effect against bacterial translocation due to disuse intestinal atrophy and bacterial overgrowth. Given the fact that only limited therapeutic options are available for SBS and its associated comorbidity of IFALD, polyamines ingestion could be readily applied in a clinical setting for its favorable safety profile^47^ and affordability. Combined administration of polyamines and GLP-2 analog would elicit an additive effect in SBS patients due to their different mechanisms. Moreover, the oral administration of polyamines acts additively rather than obstructing other existing therapeutic modalities such as surgical intestinal lengthening procedures, PN and intestinal rehabilitation program. A robust clinical trial that demonstrates the effectiveness of polyamines in SBS should be performed.

## Materials and methods

### Animals

All experiments were conducted with approval from the Animal Care Committee of Jichi Medical University (approval number: 15-213) and performed in accordance with ARRIVE guidelines and the Japanese Guidelines for Animal Research. Male Lewis rats were purchased from Charles River Laboratories (Yokohama, Japan). Rats aged 7–8 weeks old that weighed 214–263 g were used here. They were housed in a temperature- and humidity-controlled environment with a 12/12 h light/dark cycle with ad libitum access to food (CE-2; CLEA Japan Co., Ltd., Tokyo, Japan) and water.

### Surgical procedure

After fasting overnight, all procedures were performed under inhalation anesthesia using 1.5% isoflurane. The abdomen was opened through a midline incision. Two-thirds of the small intestine (from 5 cm distal of the Treitz ligament to 10 cm proximal of the ileo-cecal valve) was resected, resulting in 15 cm of remnant intestine. Anastomosis of the remnant jejunum to ileum was performed with 7–0 Maxon (Covidien Japan Inc., Tokyo, Japan). The abdomen was closed with 4–0 nylon sutures and the rats were allowed to recover from anesthesia. There was no mortality associated with this surgical procedure. After the operation, the rats were fed a standard diet (CE-2) for 3 weeks, followed by 1 week of 0% PD. Then, they were randomly allocated into three groups and fed diets with different polyamine contents for 30 days after the operation. The sham group underwent laparotomy and cut off small intestine at 5 cm distal of the Treitz ligament with reanastmosis and was fed a standard diet for 3 weeks, followed by a 0% PA diet until the end of the experiment. Animals were fasted overnight and sacrificed after 30 days of consuming the respective PD. Blood samples were taken from the inferior vena cava, and the intestine, liver, and feces were harvested. All samples were appropriately preserved for subsequent procedures.

### Rat diet

Fortified rodent diet for growth (AIN-93G; Oriental Bio Service, Tokyo, Japan) was modified into 0% polyamine, 0.01% polyamine, and 0.1% polyamine diets (Supplementary Table S1). The soybean oil contained in AIN-93G was replaced with corn oil as fat because soybean products contain high concentrations of polyamine. For the polyamine 0.01% and 0.1% diets, spermidine trihydrochloride (Spd) and spermine tetrahydrochloride (Spm) (Sigma-Aldrich, St. Louis, MO, USA) were mixed to reach the net concentrations of Spd and Spm of 0.008% and 0.002% (w/w), and 0.08% and 0.02% (w/w), respectively. The above-mentioned relative proportions of Spd and Spm were determined to be approximate to that of soybean germ. We measured the polyamine content of CE-2 to contain around 0.01% by weight (data not shown). Thus, 0.01% polyamine diet can be considered as physiologic dose in terms of polyamine content and 0.1% polyamine diet as supraphysiologic for rodents. Polyamines intake by the rats fed high polyamine diet in this experiment was estimated to be approximately several hundred of times higher per body weight compared to the daily intake by humans^48^. When this dosage is extrapolated to humans using allometric scaling, it becomes several tens of times the normal intake.

### Measurement of polyamine content in tissue and feces

To measure the concentrations of the polyamines, each intestine, whole blood, liver, and fecal sample was transferred to a new microfuge tube containing 10% trichloroacetic acid. After centrifugation at 2000 g for 15 min, each of the resulting supernatants was collected and was double-diluted with 0.1N hydrochloric acid. The concentrations of Spd, Spm, and Put were measured by high-performance liquid chromatography, in accordance with a previous report^49^.

### Fecal mucin measurement

Fecal mucins were extracted and quantitated using a fluorometric assay (Fecal Mucin Assay Kit, FFA-MU-K01; COSMO BIO Co., Ltd., Tokyo, Japan).

### Measurement of secretory IgA content in feces and serum

Feces were collected and stored at − 80 °C before analysis. In brief, they were dried for 30 min in a vacuum dryer at room temperature. Later, 0.1 g of a dried sample was weighed and vortexed in 1 ml of normal saline and centrifuged, and the supernatant was collected and centrifuged. The concentrations of secretory IgA in feces and sera were determined using Rat IgA ELISA Kit (E111-102; Bethyl Laboratories, Inc., Montgomery, TX, USA), in accordance with the manufacturer’s protocol.

### Measurement of serum diamine oxidase (DAO) and GLP-2 concentrations

To measure the concentration of serum diamine oxidase (DAO), the Diamine Oxidase ELISA Kit (KR8500; Immundiagnostik GmbH, Bensheim, Germany) was used, in accordance with the manufacturer’s protocol. Blood samples for GLP-2 analyses were collected with the BD™ P800 Blood Collection System (BD Biosciences, San Jose, CA, USA), which is an evacuated blood collection tube containing a cocktail of protease inhibitors, optimized for the stabilization of serum GLP-2. To measure the concentration of serum GLP-2, Rat GLP-2 ELISA Kit (292-60601; Wako Pure Chemical Industries, Tokyo, Japan) was used, in accordance with the manufacturer’s protocol.

### Measurement of fecal short-chain fatty acid (SCFA) content

Frozen fecal samples were sent to Technosuruga Laboratory Co. Ltd. (Shizuoka, Japan). Fecal SCFAs (seven kinds, including acetate, propionate, and n-butyrate) were extracted from the feces and measured using high-performance liquid chromatography (Prominence; Shimadzu, Kyoto, Japan), as previously described^50^. Fecal concentrations of SCFAs were expressed as mg/g of fecal wet weight.

### Histology and immunohistochemical staining

Intestine and liver tissues were fixed in 10% formalin for 24 h. Sections (4 μm) from paraffin-embedded intestine and liver tissues were subjected to hematoxylin and eosin staining for histological analysis. Villous height and crypt depth were measured blindly at a minimum of six villi or six crypts per slide using ImageJ software. For immunohistochemical staining, slides were deparaffinized, rehydrated, and treated with 3% hydrogen peroxide. Antigen retrieval was performed by boiling the sections for 20 min in 0.1 M Citrate Buffer Antigen Retrieval Solution (pH 6.0). Nonspecific antibody binding was blocked using 15% goat serum for 30 min. For staining of Ki-67, Muc2, and Claudin-3, the slides were then incubated with anti-Ki67 antibody (ab16667; Abcam, Cambridge, UK), anti-Muc2 rabbit monoclonal antibody (M01212-1; Boster Biological Technology, Pleasanton, CA, USA), and Claudin-3 polyclonal antibody (#PA5-32353; Invitrogen, Waltham, MA, USA) at 4℃ overnight, respectively. The signal was detected with the ABC kit and DAB kit (Vector Laboratories, Carlsbad, CA, USA). The sections were counterstained with hematoxylin. The average number of Ki-67-positive cells in the crypt area was expressed by dividing the number of Ki-67-positive cells by the measured crypt area in at least four different fields per slide on four animals per group. The average number of goblet cells in the villous area was calculated by dividing the number of goblet cells by the measured villous area for at least three different villi per slide on four animals per group. The average area of goblet cell granule was calculated by dividing the total goblet cell area by the measured villous area for at least three villi per slide on four animals per group.

The effects of oral polyamine intake on mitochondria in liver were examined by immunofluorescent staining. Staining for HSP60 (mitochondrial marker) was performed in accordance with a protocol for indirect immunofluorescence using TSA CYANINE 3 SYSTEM (NEL704A001KT; Akoya Biosciences, Waltham, MA, USA). After antigen retrieval and blocking nonspecific binding sites, sections were incubated with monoclonal anti-HSP60 (66041-1-Ig; Proteintech, Chicago, IL, USA) diluted in blocking buffer, followed by the introduction of HRP and signal amplification. Nuclei were counterstained with 4,6-diamidino-2-phenylindole (DAPI) diluted in PBS. Representative images were acquired on an Olympus confocal laser scanning microscope (FluoView FV1000).

### mtDNA copy number

Total DNA was isolated from liver samples using DNA Easy Extraction Kit (KN-T110005; Kaneka, Tokyo, Japan). The hepatic mtDNA copy number was assessed by determining the copy number of the mitochondrial genome-encoded ATP synthase subunit 6 gene (mt-Atp6) relative to the single-copy nuclear peroxisome proliferator-activated receptor-γ coactivator-1α (Ppargc1a) gene and the mitochondrially encoded 16S rRNA gene (mt-Rnr2) relative to the single-copy nuclear glyceraldehyde-3-phosphate dehydrogenase (GAPDH) using quantitative PCR, as previously described^51^. Real-time PCR was performed in MicroAmp optical 96-well plates using the 7500 Real-Time PCR System (Applied Biosystems, Foster City, CA, USA). The reaction volume of 15 μl contained 10 ng of genomic DNA, forward and reverse primers (0.25 μM each), and 2 × TB Green Premix Ex Taq II (RR820S; Takara Bio Inc., Kyoto, Japan). Primer sequences are listed in Supplementary Table   S2.

### Western blot assay

Western blot analyses were performed using primary antibodies recognizing IgA (A110-102AP; Bethyl Laboratories), Claudin-3 (#PA5-32353; Invitrogen), Albumin (#PA5-89332; Invitrogen), phosphorylated-AMPKα (Phospho-AMPKα; Thr172; #2535; Cell Signaling Technology, Danvers, MA, USA), LC-3 (M152-3; MBL, Tokyo, Japan), p62 (PM045; MBL), β-actin (sc-47778; Santa Cruz Biotechnology, Santa Cruz, CA, USA), and α-Tubulin (PM054-7; MBL). Goat anti-rabbit IgG H&L (HRP) (ab205718; Abcam) was used as a secondary antibody at 1:2000 dilution. The bands were detected using enhanced chemiluminescence reagent (Cytiva, Malborough, MA, USA) and the ChemiDoc MP Imaging System (Bio-Rad, Hercules, CA, USA). Results were analyzed using ImageJ.

### Measurement of liver cholesterol and triglyceride

The measurement of cholesterol and triglyceride levels in rat livers using Folch’s extraction procedure was outsourced to Immuno-Biological Laboratories Co., Ltd. (Fujioka, Japan).

### Measurement of liver ATP

The hepatic ATP concentration was measured using an ATP Colorimetric/Fluorometric Assay Kit (K354; BioVision, Inc., Milpitas, MA, USA), in accordance with the manufacturer’s protocol.

### Real-time reverse-transcription PCR

Total RNA was extracted from intestine and liver tissues using the RNeasy Plus Mini Kit (74134; Qiagen, Hilden Germany). The concentration of RNA was measured using NanoDrop1000 (Thermo Fisher, Waltman, MA, USA), followed by complementary DNA synthesis using the SuperScript™ III First-Strand Synthesis SuperMix for qRT-PCR (11752050; Thermo Fisher). The primers used for reverse-transcription PCR (RT-PCR) are listed in Supplementary Table S2. The ΔΔCt method was used to quantify the target mRNA expression using GAPDH for intestine samples and β-actin for liver samples as a reference gene.

### Statistical analysis

Prism software (GraphPad) was used for statistical analyses. Data are presented as mean ± SD. One-way ANOVA, followed by post hoc Tukey’s test (comparing the mean of each sample with the mean of every other sample), was used for multiple comparisons of normally distributed datasets with one variable. P value was used to quantify the statistical significance. *P < 0.05; **P < 0.01; ***P < 0.001; ****P < 0.0001.

### Supplementary Information

Supplementary Tables.

Supplementary Figure S1.

## Data Availability

All data generated or analysed during this study were included in this published article and its supplementary information files.

## References

[1] Oke SM (2021). Outcome of adult patients receiving parenteral support at home: 36 years' experience at a tertiary referral centre Survival of patients with SBS depends on adaptation of the remnant bowel. Clin. Nutr..

[2] Duro D, Kamin D, Duggan C (2008). Overview of pediatric short bowel syndrome. J. Pediatr. Gastroenterol. Nutr..

[3] Jeppesen PB (2014). New approaches to the treatments of short bowel syndrome associated Intestinal failure. Curr. Opin Gastroenterol..

[4] Pironi L (2011). Long-term follow-up of patients on home parenteral nutrition in Europe: Implications for intestinal transplantation. Gut.

[5] Jeppesen PB (2012). Teduglutide reduces need for parenteral support among patients with short bowel syndrome with intestinal failure. Gastroenterology.

[6] Soda K, Dobashi Y, Kano Y, Tsujinaka S, Konishi F (2009). Polyamine-rich food decreases age-associated pathology and mortality in aged mice. Exp. Gerontol..

[7] Madeo F, Eisenberg T, Pietrocola F, Kroemer G (2018). Spermidine in health and disease. Science..

[8] Eisenberg T (2009). Induction of autophagy by spermidine promotes longevity. Nat. Cell Biol..

[9] Bachmann AS, Geerts D (2018). Polyamine synthesis as a target of MYC oncogenes. J. Biol. Chem..

[10] Zou T (2006). Polyamine depletion increases cytoplasmic levels of RNA-binding protein HuR leading to stabilization of nucleophosmin and p53 mRNAs. J. Biol. Chem..

[11] Schuller AP, Wu CC, Dever TE, Buskirk AR, Green R (2017). eIF5A functions globally in translation elongation and termination. Mol. Cell..

[12] Zhou J (2022). Spermidine-mediated hypusination of translation factor EIF5A improves mitochondrial fatty acid oxidation and prevents non-alcoholic steatohepatitis progression. Nat. Commun..

[13] Okumura S (2016). Oral administration of polyamines ameliorates liver ischemia/reperfusion injury and promotes liver regeneration in rats. Liver Transpl..

[14] Doi J (2019). Bolus administration of polyamines boosts effects on hepatic ischemia-reperfusion injury and regeneration in rats. Eur. Surg. Res..

[15] Teratani T (2021). Activation of whole body by high levels of polyamine intake in rats. Amino Acids..

[16] Kamiya S (2004). The value of bile replacement during external biliary drainage: an analysis of intestinal permeability, integrity, and microflora. Ann. Surg..

[17] Liu G (2020). Digestive abilities, amino acid transporter expression, and metabolism in the intestines of piglets fed with spermine. J. Food Biochem..

[18] Atiya Ali M (2013). Polyamine levels in breast milk are associated with mothers' dietary intake and are higher in preterm than full-term human milk and formulas. J. Hum. Nutr. Diet..

[19] Larqué E, Sabater-Molina M, Zamora S (2007). Biological significance of dietary polyamines. Nutrition..

[20] Nightingale J, Woodward JM (2006). Small Bowel and Nutrition Committee of the British Society of Gastroenterology. Guidelines for management of patients with a short bowel. Gut..

[21] Mantis NJ, Rol N, Corthésy B (2011). Secretory IgA's complex roles in immunity and mucosal homeostasis in the gut. Mucosal. Immunol..

[22] Velcich A, Yang W, Heyer J, Fragale A, Nicholas C, Viani S (2002). Colorectal cancer in mice genetically deficient in the mucin Muc2. Science..

[23] Shim S (2015). Claudin-3 expression in radiation-exposed rat models: A potential marker for radiation-induced intestinal barrier failure. Biochem. Biophys. Res. Commun..

[24] Corthésy B (2013). Multi-faceted functions of secretory IgA at mucosal surfaces. Front. Immunol..

[25] Wells JM, Rossi O, Meijerink M, van Baarlen P (2011). Epithelial crosstalk at the microbiota-mucosal interface. Proc. Natl. Acad. Sci. U.S.A..

[26] Duangnumsawang Y, Zentek J, Goodarzi Boroojeni F (2021). Development and functional properties of intestinal mucus layer in poultry. Front. Immunol..

[27] Garcia-Hernandez V, Quiros M, Nusrat A (2017). Intestinal epithelial claudins: Expression and regulation in homeostasis and inflammation. Ann. N.Y. Acad. Sci..

[28] Liu L (2009). Polyamines regulate c-Myc translation through Chk2-dependent HuR phosphorylation. Mol. Biol Cell..

[29] Liu P (2021). The role of short-chain fatty acids in intestinal barrier function, inflammation, oxidative stress, and colonic carcinogenesis. Pharmacol. Res..

[30] Drucker DJ, Erlich P, Asa SL, Brubaker PL (1996). Induction of intestinal epithelial proliferation by glucagon-like peptide 2. Proc. Natl. Acad. Sci. U.S.A..

[31] Guan X (2006). GLP-2 receptor localizes to enteric neurons and endocrine cells expressing vasoactive peptides and mediates increased blood flow. Gastroenterology..

[32] Drucker DJ, Yusta B (2014). Physiology and pharmacology of the enteroendocrine hormone glucagon-like peptide-2. Ann. Rev. Physiol..

[33] Mutanen A, Pakarinen MP (2017). Serum fasting GLP-1 and GLP-2 associate with intestinal adaptation in pediatric onset intestinal failure. Clin. Nutr..

[34] Lee WS, Chew KS, Ng RT, Kasmi KE, Sokol RJ (2020). Intestinal failure-associated liver disease (IFALD): Insights into pathogenesis and advances in management. Hepatol. Int..

[35] Calkins KL (2014). Pediatric intestinal failure-associated liver disease is reversed with 6 months of intravenous fish oil. JPEN J. Parenter. Enteral. Nutr..

[36] Mundi MS, Kuchkuntla AR, Salonen BR, Bonnes S, Hurt RT (2020). Long-term use of mixed-oil lipid emulsion in soybean oil-intolerant home parenteral nutrition patients. JPEN J. Parenter. Enteral. Nutr..

[37] Kaur J, Debnath J (2015). Autophagy at the crossroads of catabolism and anabolism. Nat. Rev. Mol. Cell Biol..

[38] Takagi A, Kume S, Maegawa H, Uzu T (2016). Emerging role of mammalian autophagy in ketogenesis to overcome starvation. Autophagy..

[39] Zhang H (2019). Polyamines control eIF5A hypusination, TFEB translation, and autophagy to reverse B cell senescence. Mol. Cell..

[40] Yoo J, Jeong IK, Ahn KJ, Chung HY, Hwang YC (2021). Fenofibrate, a PPARα agonist, reduces hepatic fat accumulation through the upregulation of TFEB-mediated lipophagy. Metabolism..

[41] van Zutphen T (2016). Malnutrition-associated liver steatosis and ATP depletion is caused by peroxisomal and mitochondrial dysfunction. J. Hepatol..

[42] Sookoian S (2010). Epigenetic regulation of insulin resistance in nonalcoholic fatty liver disease: Impact of liver methylation of the peroxisome proliferator-activated receptor γ coactivator 1α promoter. Hepatology..

[43] Onishi S (2016). The administration of ghrelin improved hepatocellular injury following parenteral feeding in a rat model of short bowel syndrome. Pediatr. Surg. Int..

[44] Machigashira S (2018). The protective effect of fish oil lipid emulsions on intestinal failure-associated liver disease in a rat model of short-bowel syndrome. Pediatr. Surg. Int..

[45] Yano K (2019). Novel effect of glucagon-like peptide-2 for hepatocellular injury in a parenterally fed rat model of short bowel syndrome. Pediatr. Surg. Int..

[46] Yano K (2022). The preventive effect of recombinant human hepatocyte growth factor for hepatic steatosis in a rat model of short bowel syndrome. J. Pediatr. Surg..

[47] Muñoz-Esparza NC (2019). Polyamines in food. Front. Nutr..

[48] Ali MA, Poortvliet E, Strömberg R, Yngve A (2011). Polyamines: Total daily intake in adolescents compared to the intake estimated from the Swedish Nutrition Recommendations Objectified (SNO). Food Nutr. Res..

[49] Saito K, Horie M, Nose N, Nakagomi K, Nakazawa H (1992). Determination of polyamines in foods by liquid chromatography with on-column fluorescence derivatization. Anal. Sci..

[50] Saji N (2020). Relationship between 284 dementia and gut microbiome-associated metabolites: A cross-sectional study in Japan. Sci. Rep..

[51] Carabelli J (2011). High fat diet-induced liver steatosis promotes an increase in liver mitochondrial biogenesis in response to hypoxia. J Cell Mol. Med..

