# Supplementary material for: Dietary polyamines promote intestinal adaptation in an experimental model of short bowel syndrome
Source: Sci Rep. 2024 Feb 26;14:4605. doi: 10.1038/s41598-024-55258-4 (PMC10897130; doi:10.1038/s41598-024-55258-4)
Supplement: Supplementary file 1 — Supplementary Tables. [file 41598_2024_55258_MOESM1_ESM.docx]

**Supporting information**

**Supplementary Table S1.** Complete ingredients and nutritional contents of AIN-93G and the three kinds of test diet used in the experiment.

| Ingredients | AIN-93G | 0% polyamine diet | 0.01% polyamine diet | 0.1% polyamine diet |
| --- | --- | --- | --- | --- |
| Casein | 20.00 | 20.00 | 20.00 | 20.00 |
| L-Cystin | 0.30 | 0.30 | 0.30 | 0.30 |
| Corn starch | 39.75 | 39.75 | 39.75 | 39.75 |
| Pregelatinized corn starch | 13.20 | 13.20 | 13.20 | 13.20 |
| Sucrose | 10.00 | 10.00 | 10.00 | 10.00 |
| Refined soybean oil | 7.00 | － | － | － |
| Corn oil | － | 7.00 | 7.00 | 7.00 |
| Cellulose | 5.00 | 5.00 | 5.00 | 5.00 |
| AIN93G mineral mix | 3.50 | 3.50 | 3.50 | 3.50 |
| AIN93 vitamin mix | 1.00 | 1.00 | 1.00 | 1.00 |
| Choline bitartrate | 0.25 | 0.25 | 0.25 | 0.25 |
| Tertiary butylhydroquinone | 0.0014 | 0.0014 | 0.0014 | 0.0014 |
| SPD trihydrochloride | － | － | 0.0146 (0.008) | 0.146(0.08) |
| SPM tetrahydrochloride | － | － | 0.0036 (0.002) | 0.036(0.02) |
| Values in parentheses are the compounding ratio (w/w, %). | | | | |

**Supplementary Table S2.** Real-time qPCR primer sequences used in this study.

| Genes | Sequence (forward 5'-3') | Sequence (reverse 3'-5') | gene ID |
| --- | --- | --- | --- |
| GAPDH | GGCACAGTCAAGGCTGAGAATG | ATGGTGGTGAAGACGCCAGTA | 24383 |
| β-actin | GGAGATTACTGCCCTGGCTCCTA | GACTCATCGTACTCCTGCTTGCTG | 81822 |
| SI | AGCCAACGGTGCAGAATATC | GTTAATCCTGGCCATACCTCTC | 497756 |
| Vil1 | TGTAGTCCTGGCTATCCACA | CAGGTAGTCATCCATCTGTGTT | 316521 |
| Sox9 | CAAGAACAAGCCACACGTCA | CTCTCGTTCAGCAGTCTCCA | 140586 |
| DPP4 | ACAGAATCTCGTTGCAGTGG | AATATGCTCCTGCGTCGTTG | 25253 |
| GLUT5 | GCAACGATGGAGAAAGAGGAT | GAACTGCTGCATGAACTCTGA | 65197 |
| SGLT1 | CTTCGAATGGAACGCCTTGG | AATCGCTTCCGCAGATACTC | 25552 |
| Slc3a2 | CTCCTCAGGCTGACATTGTA | AGTACAAGGATGCATTCGCC | 50567 |
| Slc15a1 | ACTGGAGTTCTCCTATTCCCA | CATACTCAGCCCACTGTTTGT | 117261 |
| APOA1 | AAGGACAGCGGCAGAGACTA | AACCCAGAGTGTCCCAGTTG | 25081 |
| FABP1 | CGAACTGGAGACCATGACTG | CCAGTGTCATGGTATTGGTGA | 24360 |
| FABP2 | GCAGATGGAACAGAACTCACT | CTCCTTCATATGTGTAGGTTTGGA | 25598 |
| Gstm1 | TTCGTGCAGACATTGTGGAGA | CTTGCCCAGGAACTCAGAGTAGA | 24423 |
| LXRα | CGTGCAGGAGATTGTTGACT | TCCAGAAGCATCACCTCGAT | 58852 |
| SREBP-1c | GGTACCTGCGGGACAGCTTA | GGCTGAAGCTGCTGACTGTTG | 78968 |
| SREBP-2 | CAACTGTTCGACTAATGGCG | GGTGTCTACTTCTCCGTGTT | 300095 |
| ACC1 | TCACCATCAGCCTGGTTACA | CCGTTGTTGTGCATTATCTGG | 60581 |
| FAS | GCTGCTACAAACAGGACCATCAC | TCTTGCTGGCCTCCACTGAC | 50671 |
| Elovl6 | GGTCGGCATCTGATGAACAA | CGAAGAGCACCGAATATACTGA | 171402 |
| CD36 | CCAGACAACCACTGTTTCTGCAC | CATCTTCGTTAGGATTCAAGCCTTC | 29184 |
| Cpt1a | CTGCCAGTTCCATTAAGCCACA | CAGCTATGCAGCCTTTGACTACCA | 25757 |
| Tfam | TGAAGCTTGTAAATCAGGCTTGGA | CATCTTCGTTAGGATTCAAGCCTTC | 83474 |
| Abca1 | GTGTTCTTCCTCGTTACGGT | GCTTCCTTCTGTAGATCTTGGT | 313210 |
| G6PD | GCTGGACCTAACCTATGGCAACA | GTGCATTTGGCTCCCACAGA | 24377 |
| Pgd | AGGCTTTATGCTGCTCAGACAGG | GGGTTTCGCTCAAAGGCATC | 100360180 |
| mt-Atp6 | ACACCAAAAGGACGAACCTG | ATGGGGAAGAAGCCCTAGAA | 26197 |
| nPpargc1a | ATGAATGCAGCGGTCTTAGC | AACAATGGCAGGGTTTGTTC | 83516 |
| mt-Rnr2 | AGCTATTAATGGTTCGTTTGT | AGGAGGCTCCATTTCTCTTGT | 170603 |
| nGAPDH | GGAAAGACAGGTGTTTTGCA | AGGTCAGAGTGAGCAGGACA | 24383 |

GAPDH: glyceraldehyde-3-phosphate dehydrogenase; SI: sucrase-isomaltase; Vil1: villin 1; Sox9: SRY-box transcription factor 9; DPP4: dipeptidyl peptidase 4; GLUT5: glucose transporter-like protein 5; SGLT1: sodium-dependent glucose transporter 1; Slc3a2: solute carrier family 3 member 2; Slc15a1: solute carrier family 15 member 1; APOA1: apolipoprotein A1; FABP1: fatty acid binding protein 1; FABP2: fatty acid binding protein 2; Gstm1: glutathione S-transferase Mu 1; LXRα: liver X receptor alpha; SREBP-1c: sterol regulatory element-binding protein 1c; SREBP-2: sterol regulatory element-binding protein 2; ACC1: acetyl-CoA carboxylase 1; FAS: fatty acid synthase; Elovl6: elongation of very long chain fatty acids protein 6; Cpt1a: carnitine palmitoyltransferase 1A; Tfam: transcription factor A, mitochondrial; Abca1: ATP binding cassette subfamily A member 1; G6PD: glucose-6-phosphate dehydrogenase; Pgd: phosphogluconate dehydrogenase; mt-Atp6: mitochondrially encoded ATP synthase 6; Ppargc1a: Peroxisome proliferator-activated receptor gamma coactivator 1-alpha; mt-Rnr2: mitochondrially encoded 16S rRNA
