# Supplementary material for: Dietary polyamines promote intestinal adaptation in an experimental model of short bowel syndrome
Source: Sci Rep. 2024 Feb 26;14:4605. doi: 10.1038/s41598-024-55258-4 (PMC10897130; doi:10.1038/s41598-024-55258-4)
Supplement: Supplementary file 2 — Supplementary Figure S1. [file 41598_2024_55258_MOESM2_ESM.pdf]

Supplementary Information FigureS1. Representative gel images of western blot analyses.

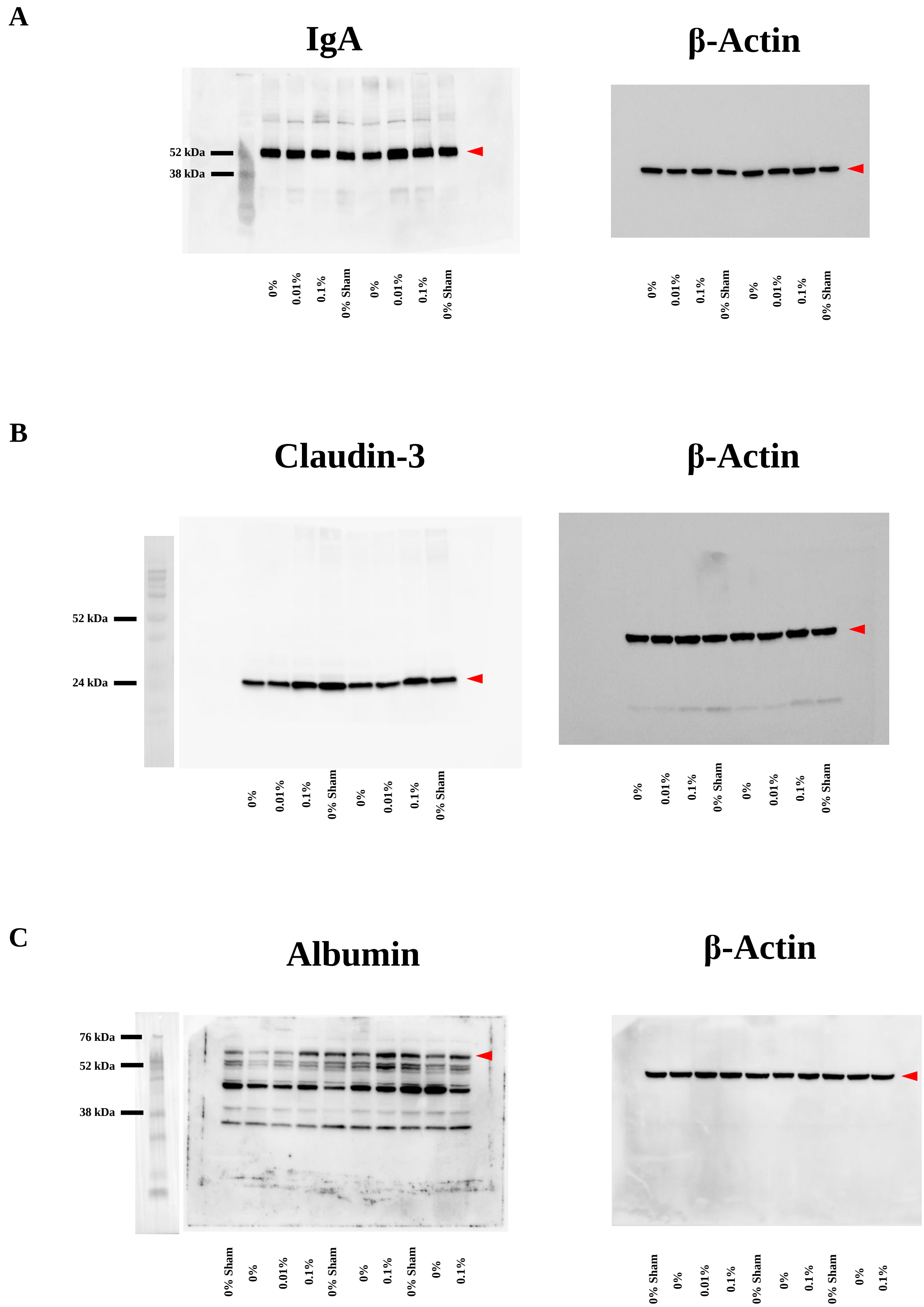

D

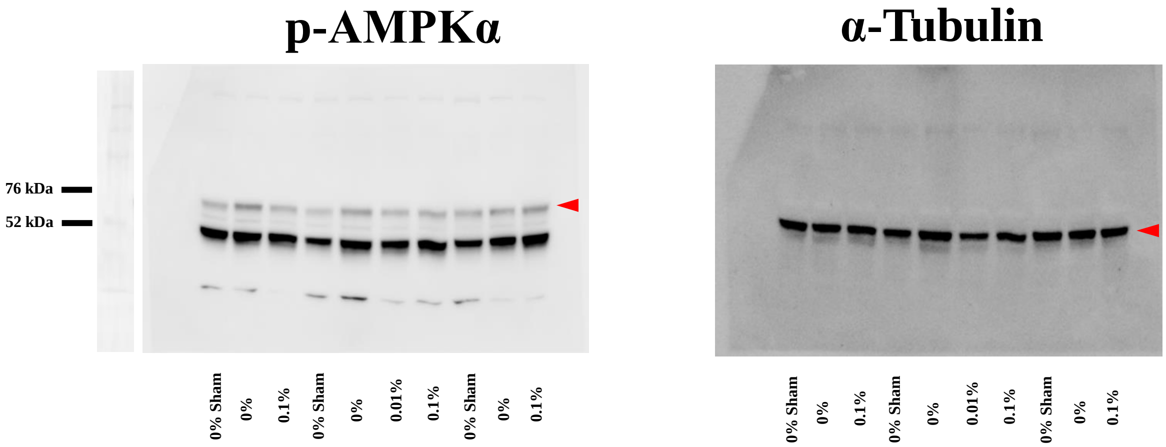
p-AMPKα
α-Tubulin
76 kDa
52 kDa
0% Sham
0%
0.1%
0% Sham
0%
0.01%
0.1%
0% Sham
0%
0.1%

E

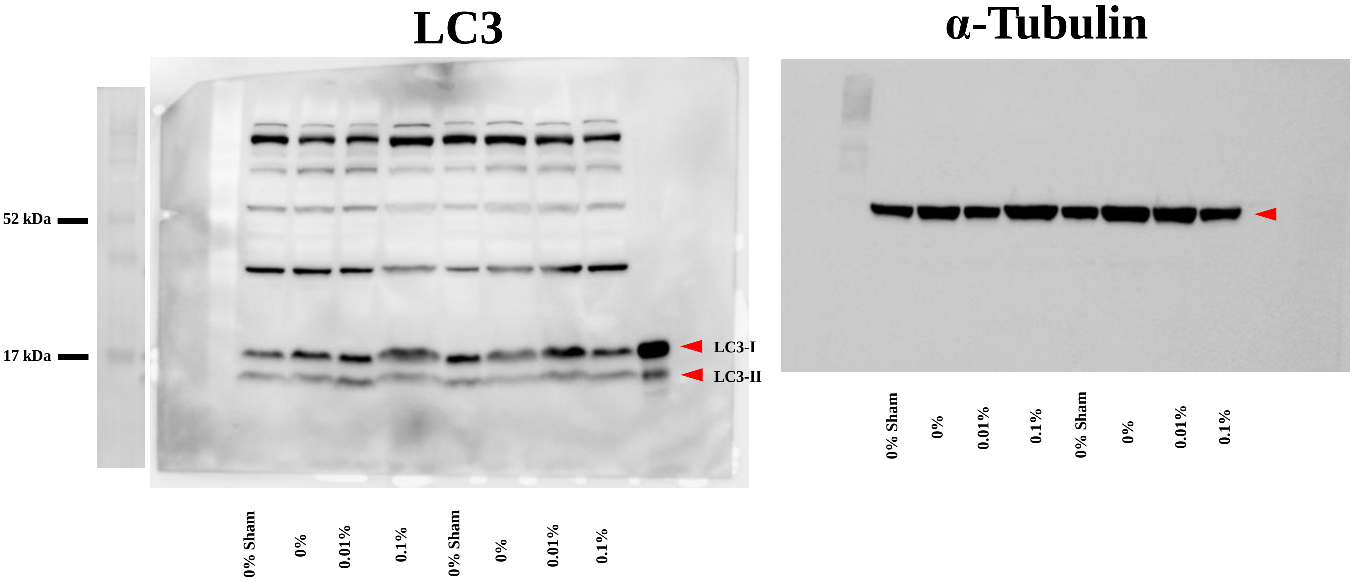
LC3
α-Tubulin
52 kDa
17 kDa
LC3-I
LC3-II
0% Sham
0%
0.01%
0.1%
0% Sham
0%
0.01%
0.1%

F

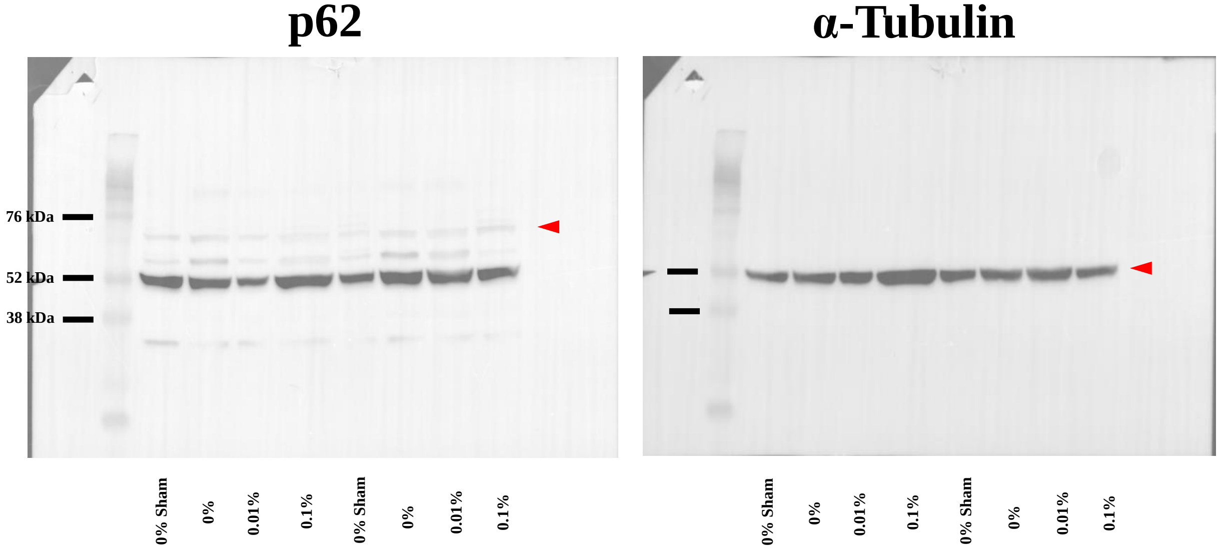
p62
α-Tubulin
76 kDa
52 kDa
38 kDa
0% Sham
0%
0.01%
0.1%
0% Sham
0%
0.01%
0.1%
